# Addressing the Misuse Potential of Life Science Research—Perspectives From a Bottom-Up Initiative in Switzerland

**DOI:** 10.3389/fbioe.2018.00038

**Published:** 2018-04-05

**Authors:** Franziska M. Oeschger, Ursula Jenal

**Affiliations:** ^1^Forum for Genetic Research, Swiss Academy of Sciences, Bern, Switzerland; ^2^Jenal & Partners Biosafety Consulting, Rheinfelden, Switzerland

**Keywords:** biosecurity, dual use research, misuse, science policy, community engagement, risk assessment, risk management, research regulation

## Abstract

Codes of conduct have received wide attention as a bottom-up approach to foster responsibility for dual use aspects of life science research within the scientific community. In Switzerland, a series of discussion sessions led by the Swiss Academy of Sciences with over 40 representatives of most Swiss academic life science research institutions has revealed that while a formal code of conduct was considered too restrictive, a bottom-up approach toward awareness raising and education and demonstrating scientists' responsibility toward society was highly welcomed. Consequently, an informational brochure on “Misuse potential and biosecurity in life sciences research” was developed to provide material for further discussions and education.

## Introduction

### Background

Since the 2001 anthrax attacks in the US, prevention of biocrimes and bioterrorism has been a concern of governing bodies worldwide. Strengthening oversight of research in the life sciences is one key element considered in this context (National Science Advisory Board on Biosecurity, 2007; United Nations Office at Geneva, 2008; World Health Organisation, 2010). Oversight mechanisms need to be balanced to offer effective protection against risks of misuse while at the same time guaranteeing freedom of research and allowing much-needed research that addresses pressing societal and environmental challenges. Proposed and in some places already implemented oversight measures range from legally binding, government-led top-down approaches (e.g., moratorium; export licenses for scientific publications; national biosecurity laws) to bottom-up self-regulatory initiatives (e.g., codes of conduct; awareness-raising activities). In Europe, national legislations differ widely in terms of specificity and restrictiveness, with Denmark representing one of the first countries to put a specific biosecurity law into force (Uhlenhaut et al., 2013; Harris, 2016). In parallel, expert bodies worldwide have suggested that bottom-up awareness-raising initiatives could replace or complement top-down approaches by sensitizing individual researchers and research institutions to risks (National Research Council, 2004; National Science Advisory Board on Biosecurity, 2007; United Nations Office at Geneva, 2008; Stroot and Jenal, 2011).

### Codes of conduct

Codes of conduct have received wide consideration as an appropriate measure to raise awareness of and foster responsibility for dual use aspects of life science research within the scientific community (United Nations Office at Geneva, 2005; National Science Advisory Board on Biosecurity, 2007; World Health Organisation, 2010; Royal Netherlands Academy of Arts and Sciences, 2013). A code of conduct intends to promote ethical principles and corresponding behavioral norms that often go beyond legal requirements. Several national and international institutions have developed codes of conducts addressing the misuse potential of life science research. Others have incorporated requirements in more comprehensive codes of ethics. We analyzed eight such documents that all specifically address the misuse potential of biological research (Table 1). Many of these codes base themselves on the guiding principle that life scientists have an ethical obligation to prevent or minimize risk and harm that could result from research outcomes, including the malevolent misuse by others. To support this principle, the following behavioral rules are proposed:

- Be aware of the misuse potential of your own research and assess your research projects routinely
- Modify your research to reduce risks
- Report and document risks
- Know and follow regulations, guidelines and safe practices
- Protect sensitive material and data
- Educate and train others and act as a role model

**Table 1:** Selection of guidance documents and codes addressing the misuse potential of biological research.

**Authors**	**Title**	**Year**
InterAcademy Panel (IAP)	IAP statement on biosecurity	2005
International Union of Microbiological Societies (IUMS)	IUMS code of ethics against misuse of scientific knowledge, research and resources	2005
U.S. National Science Advisory Board for Biosecurity (NSABB)	Considerations in developing a code of conduct for dual use research in the life sciences	2007
Royal Netherlands Academy of Arts and Sciences (KNAW)	A code of conduct for biosecurity	2008
Comitato Nazionale per la Biosicurezza, le Biotecnologie e le Scienze della Vita	Codice di condotta per la biosicurezza	2010
Max Planck Society	Guidelines and rules of the Max Planck Society on a responsible approach to freedom of research and research risks	2010
Deutsche Forschungsgemeinschaft (DFG)	Verhaltenskodex: Arbeit mit hochpathogenen Mikroorganismen und Toxinen	2013
BBSRC, MRC, Wellcome Trust	Position statement on dual use research of concern and research misuse	2015

In addition, some of the documents also propose rules that might be viewed as more controversial. For instance, the Max Planck Society and the Royal Netherlands Academy of Arts and Sciences (KNAW) propose to consider in some cases modifying communication of research results in order to minimize risks, while in contrast, the Deutsche Forschungsgemeinschaft emphasizes the importance of unrestricted communication for the scientific system. Although a number of codes propose that research should be modified to reduce risks, one code includes the proposal to go beyond modification all the way to entirely refraining from research with disproportionate risks, even when it is legal (Max Planck Society). Another more contested point could be the proposed obligation to raise concerns about suspicions of misuse (KNAW).

## Aims and approaches

Switzerland has strong research activities in life sciences, both in academia and in industry. This is for instance reflected in a consistently high *per capita* output of biotechnology research publications and patents (Forum for Genetic Research, 2014; Alexakis et al., 2015). Research with harmful pathogens (biosafety levels 3 or 4) is currently conducted in over 40 public and private research institutions^1^.

Following the intense international debate sparked by the gain-of-function experiments on H5N1 in 2012 (Casadevall and Shenk, 2012; Drenth, 2012), the Swiss Academy of Sciences' Forum for Genetic Research engaged on a bottom-up initiative to address the misuse potential of life science research together with the scientific community. The aim of the project was to develop a code of conduct that has broad support and builds upon existing national and international regulations and guidelines. We expected that developing and disseminating such a code would sensitize the Swiss life science community to biosecurity aspects of their work and foster further discussions. Setting a code of conduct could also contribute to building society's trust in research.

**Figure 1:**
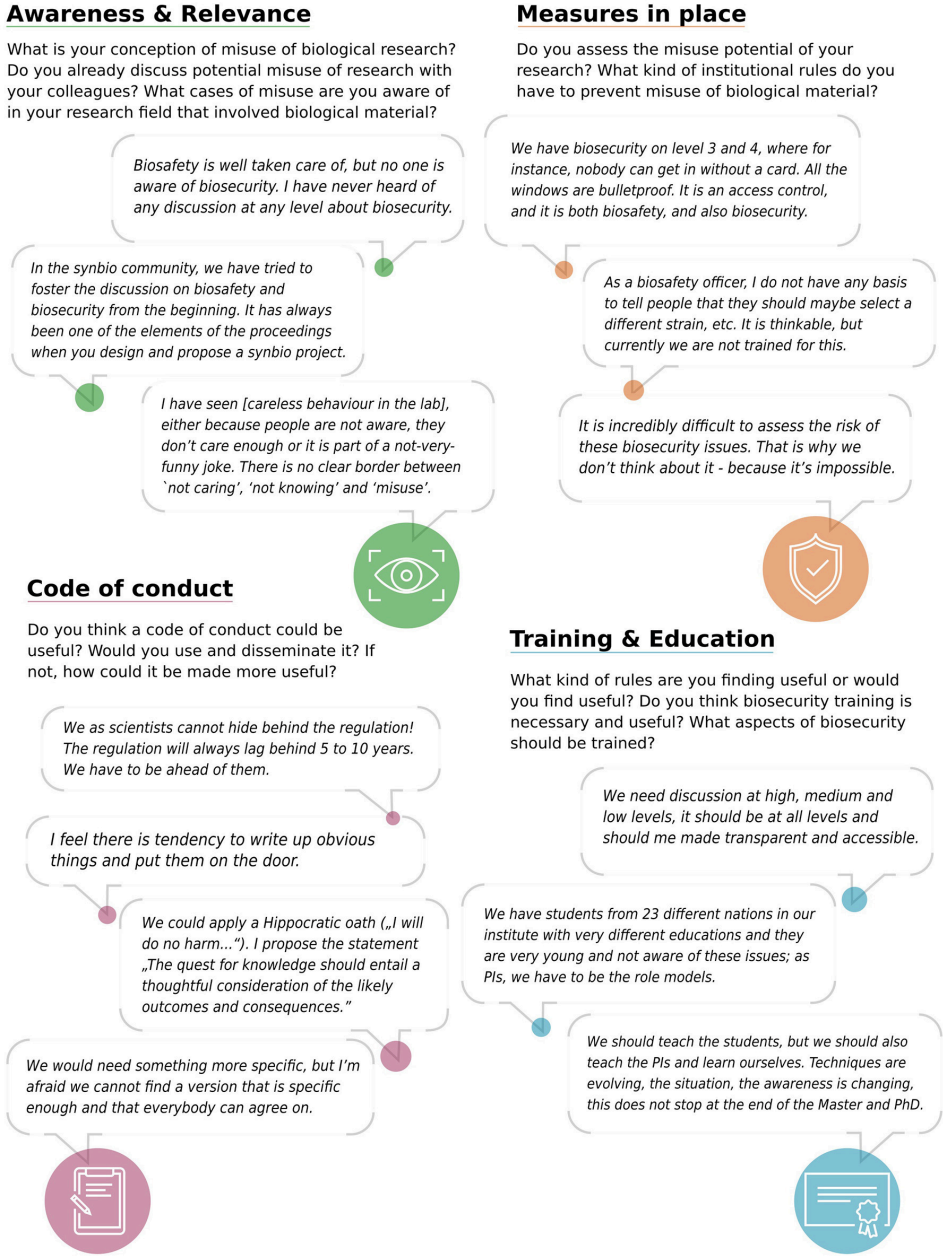
Selection of questions asked during the discussion sessions and illustrative statements from participants in respect to the different topics addressed.

As part of the initiative, life scientists were invited to share their perspectives on biosecurity aspects of their work at three structured discussion sessions and one panel discussion^2^. Efforts were made to engage life scientists from a broad range of research fields into the discussion as a potential for misuse might emerge from virtually all research fields that work with biological material and develop and apply related technologies. With these discussions, we wanted to (1) get a better understanding of what relevance Swiss academic life scientists currently attribute to biosecurity topics (e.g., awareness, individual or institutional biosecurity measures in place); and (2) more specifically gauge the support for a code of conduct (see Figure 1 for details).

In total, 46 scientists at various career stages (28 senior scientists; 12 postdocs/graduate students; 4 research support staff; 2 non-academic scientists) participated in the discussions. Around a quarter of the participants were conducting research in virology or bacteriology while the others represented a wide range of topics including molecular and cellular biology, immunology, synthetic biology, pharmacology, nanobiology, genomics, neurosciences and plant biology. Some of the sessions were joined by humanities scientists (ethics, philosophy) and members of the Swiss “Do-it-yourself-Biology” community who added additional perspectives.

## Results

### Awareness

The awareness of the dual use nature of life science research varied strongly with the specific type of research individual participants were involved in. Not surprisingly, scientists working with harmful infectious agents (e.g., influenza virus, prions) as well as some researchers in synthetic biology expressed the greatest awareness. They described considerations on biosecurity as already inherent to the design and execution of their research and as a topic of recurring discussions in their laboratories and among colleagues. Outside of these fields, however, participants estimated the awareness for the dual use nature of biological research as low—not only among their peers and students but also among biosafety officers and institute management. In the same line, graduate students reported that discussions on the misuse potential of biological research had not been part of their university education even when related topics such as scientific integrity and biosafety had been covered.

### Voluntary measures

Among the over 30 different academic life science institutes represented, only one was reported to have put in place specific guidelines for responsible behavior. Similarly, the “Do-it-yourself-Biology” community had developed a code of ethics for their members. Participants stressed, however, that their institutes and laboratories were following strict biosafety measures, which to a significant extent also cover biosecurity concerns.

It is noteworthy, that since the completion of this project, the Spiez Laboratory—the Swiss Federal Institute for NBC (nuclear, biological, chemical) protection—has issued a dual use code of conduct for its employees^3^. To our knowledge, this is the first code of conduct specifically addressing dual use aspects of research at a Swiss public institution conducting life science research.

### Relevance

Only one participant reported having been involved in a case where conscious misuse of biological material was suspected, and another having declined a request for international collaboration on the grounds of a misuse risk. Asked for a more general evaluation, most participants estimated the risk of misuse of biological material as very low in comparison with other criminal or terrorist threats. It was put forward that even though many molecular and biotechnological tools had been available for years, only one large-scale incidence of research-related misuse has occurred so far, i.e., the Anthrax letters in 2001. It was further considered as highly unlikely that individual criminals or terrorist groups would turn to cutting-edge biotechnologies (such as genome editing tools or synthetic biology) or novel research data (such as DNA sequences from gain-of-function experiments) to develop bioweapons, especially as natural pathogens are easily accessible in the environment or medical facilities. However, the worry that rogue state governments might make use of life science technologies and results in secret bioweapons programs was expressed by at least some participants. Importantly, for most researchers, estimating the risk as very low didn't preclude the need for further discussions of the topic.

### Support for a code of conduct

Most participants shared the belief that scientists had a responsibility toward society that goes beyond legal requirements. They also strongly expressed the opinion that misuse of life science research is a topic that needs to be addressed by the life science research community itself as proper risk evaluation and management depends on expert knowledge. Correspondingly, most scientists questioned the use of implementing biosecurity regulations at a national level.

A majority of participants also questioned the usefulness of a code of conduct. When shown a compilation of the content of existing codes (listed in Table 1, see also Supplementary Material), many criticized the restrictive and negative tone. Instead of prescribing behavioral norms, some would have preferred a document that states fundamental values and supports researchers' reflection about the aim and potential of their project. In contrast, others would have liked to see more concrete and specific rules and advice. It was conceded, though, that the later would be difficult to achieve for a code of conduct destined at all life scientists and that specific rules would be more likely have to be developed by individual research institutions.

Many scientists also questioned whether and how such a code could be effectively implemented and enforced. In place of a code of conduct, some suggested to develop a one-sentence statement affirming scientists' commitment to take responsibility toward the good for society while designing and doing life-science research. Such a statement would be comparable to the Hippocratic Oath to which medical professionals are bound.

### Awareness raising and education

Awareness raising and continuous open discussions within the broad scientific community were almost unanimously seen as the most effective and adequate measures to address the misuse potential of life science research. To support such efforts, many participants expressed interest in a non-binding document such as an informational brochure as a starting point. In additions, most participants would welcome training opportunities for students, researchers, research support staff and management as well as educational material. For a few participants, however, such measures wouldn't go far enough. They pointed out that the current system of evaluating research and the competitive environment do not favor pro-active communication of risks and uncertainties that go beyond legal requirements. It was thus proposed that research funders should invite more critical self-evaluations during the grant application process, for instance by awarding additional funding for projects that explicitly address and research biosecurity aspects.

In addition, it was considered as very important that researchers demonstrated their sense of responsibility toward society: through open communication of concerns about the misuse potential of life science research and through information about biosecurity measures that are being taken to prevent such misuse.

## Conclusions and perspectives

Discussions with over 40 members of the Swiss academic life sciences community revealed that overall the awareness for the misuse potential of biological research is limited, except among researchers working with dangerous infectious agents or synthetic biology. Only a small minority of researchers reported that they routinely considered and discussed biosecurity aspects of their work and only one research institute was reported to have put in place guidelines for responsible behavior that go beyond standard biosafety and ethics regulations. Most participants, however, shared the opinion that researchers have a responsibility toward society that goes beyond legal requirements. They were also convinced that life scientists themselves were best suited to evaluate and address biosecurity aspects of their work. Consequently, they were skeptical toward any type of formal controls, both in the form of top-down (e.g., national biosecurity law) and bottom-up measures (e.g., code of conduct). In place of a code of conduct, it was suggested to develop a one-sentence statement affirming scientists' commitment to take responsibility toward the good for society—comparable to the Hippocratic Oath. Initiatives that foster awareness for and discussions on the misuse potential as well as more generally a research environment of transparency, openness and responsibility were seen as the most appropriate and effective measure to address the dual use nature of life science research.

In response to these results, the Swiss Academy of Sciences decided to compile and publish an informational brochure that can be used as a written basis for further discussions within the scientific community (Swiss Academies of Arts and Sciences, 2017). The brochure highlights six issues that should be considered when designing, conducting, and communicating research projects and illustrates each issue with examples from actual research projects. The brochure is freely available in three national languages of Switzerland and in English^4^.

## Author contributions

UJ conceived the project and carried it out together with FO. UJ and FO analyzed the results and prepared the manuscript.

### Conflict of interest statement

The authors declare that the research was conducted in the absence of any commercial or financial relationships that could be construed as a potential conflict of interest.

## Footnotes

^1^https://www.bafu.admin.ch/bafu/en/home/state/indicators.html (Accessed Oct 16 2017).

^2^https://naturalsciences.ch/organisations/geneticresearch/topics/biosecurity (Accessed Oct 16 2017).

^3^https://www.labor-spiez.ch/pdf/en/fue/Dual-Use-Code-of-Conduct-eng.pdf (Accessed Oct 16 2017).

^4^http://akademien-schweiz.ch/en/index/Publikationen/Swiss-Academies-Reports.html (Accessed Oct 16 2017).

## Supplementary material

Supplementary Material 1. Code of conduct proposed by the Swiss Academy of Sciences at the discussion sessions based on existing guidance documents (see Table 1). It was presented to the participants of the discussion sessions and served as a basis to explore the usefulness of such a document for the Swiss academic life sciences community.
