# Supplementary material for: Addressing the Misuse Potential of Life Science Research—Perspectives From a Bottom-Up Initiative in Switzerland
Source: Front Bioeng Biotechnol. 2018 Apr 5;6:38. doi: 10.3389/fbioe.2018.00038 (PMC5895727; doi:10.3389/fbioe.2018.00038)
Supplement: Supplementary file 1 [file DataSheet1.PDF]

Supplementary Material

Addressing the misuse potential of life science research – perspectives from a bottom-up initiative in Switzerland

Franziska M Oeschger, Ursula Jenal*

*** Correspondence:** Ursula Jenal ursula.jenal@jenalpartners.ch

1 Supplementary Data

The following provisional code of conduct was proposed for discussion at the sessions organized by the Swiss Academy of Sciences. It was developed based on existing codes and guidance documents (see Table 1).

Code of conduct

1. **Awareness**

As a scientist I am aware that life science research can entail the risk of a deliberate misuse of biological materials as well as data, knowledge and technologies related to them. I recognize that individual good intentions do not always preclude the possibility of malevolent use by others.

2. Assessment of misuse potential

I assess and monitor the potential for misuse of my research during the entire execution of my project and continue to do so after its completion. I seek advice from competent persons if uncertain about the implication of my research. If substantial concerns about the potential for misuse emerge, I discuss with my institution appropriate strategies to address these concerns.

3. **Management of misuse potential**

I strive to design my research securely so as to allow for beneficial scientific advances while minimizing elements of concern about potential misuse. I include strategies to manage and mitigate the potential of misuse.

4. **Safe and secure practices**

I respect safe and secure laboratory practices appropriate for my type of research. I follow relevant regulatory requirements and international guidelines.

5. **Information transfer and publication**

I assess options for communicating research results already at the start of my project. When discussing, presenting or publishing results with a potential for misuse, I consider strategies to reduce the misuse potential and seek advice from my institution.

6. **Education and oversight**

I seek to raise awareness of the potential of misuse of life science research and to disseminate information aimed to prevent potential misuse, especially among my collaborators and those under my supervision. I am alert to potential misuse of research and act on findings or suspicion of misuse.
